# Editorial: Living Well After Organ Transplantation

**DOI:** 10.3389/ti.2025.15345

**Published:** 2025-08-25

**Authors:** Coby Annema, Kevin Fowler, Allison Jaure, Fabienne Dobbels, Sabina De Geest

**Affiliations:** ^1^ Department of Health Sciences, Section of Nursing Science, University Medical Center Groningen, University of Groningen, Groningen, Netherlands; ^2^ The Voice of the Patient, Inc., Saint Louis, MO, United States; ^3^ School of Public Health, The University of Sydney, Sydney, NSW, Australia; ^4^ Department of Public Health and Primary Care, Academic Center for Nursing and Midwifery, KU Leuven, Leuven, Belgium; ^5^ Department of Public Health, Institute of Nursing Science, University of Basel, Basel, Switzerland

**Keywords:** organ transplant recipients, life participation, wellbeing, quality of life, transplant care

Solid organ transplantation can offer many patients with end-stage organ failure improved survival and better quality of life compared to pretransplant. For transplant recipients, being able to participate in meaningful activities of life is a critically important outcome after transplantation [1–3]. However, complications, co-morbidities, medication side-effects and treatment burden can impair physical, mental, and social outcomes, which in turn might undermine a recipient’s capability to “live well” with their transplant.

Physicians, nurses, and allied health professionals have a key role in supporting transplant recipients in managing their physical and psychosocial health. However, addressing quality of life in transplant recipients remains a clinical challenge. With this special issue, we draw attention to under-investigated aspects of the quality of life of transplant recipients and highlight interventions and innovative care models that may have potential to improve quality of life and related outcomes in transplant recipients.

First, to support transplant recipients in being able to “live well,” it is important to understand the perspectives of transplant recipients on what good quality of life means to them. Therefore, the first section of this special issue is dedicated to the patient’s voice, in which three transplant recipients shared their views on how care should be provided to support patients to live well after transplant (Fowler; Sipma et al.; Schneider et al.). From diverse backgrounds (advocacy, business, and dietetics), all three recipients call for an integrated and person-centered care approach and urge transplant providers to not only focus on medical aspects but to take all aspects of transplant recipients’ daily life into consideration. Several suggestions were made to accomplish this goal, e.g., by integrating the patient’s voice into regular care, by using patient-reported outcome measures (PROMs), by adopting new models of care, or by establishing clear guidelines regarding integrated supportive care. Moreover, it was advocated that transplant recipients should see themselves as drivers of their own wellbeing by taking control over their own life, while transplant professionals take on a role of supporting better self-management and facilitating access to relevant healthcare services and programs.

The authors of the other manuscripts in this special issue endorse this plea as that healthcare professionals should strive for person-centered care and the integration of tailored interventions to support psychosocial and behavioral dimensions of transplant recipients care pathway. However, some key initiatives need to be taken to better align the needs and capabilities of transplant recipients with the type of care being provided by transplant professionals. More specifically, transplant professionals need insight into the prevalence and associated factors of key health issues after transplantation before appropriate interventions can be implemented. For instance, Hoteit et al. examined excessive daytime sleepiness, which was present in 12.7% of kidney transplant recipients. This was associated with Diabetes Mellitus and obesity and had a negative effect on recipients’ physical functioning. Hence, measuring daytime sleepiness or other sleep-related problems in transplant recipients is relevant and can be achieved through using appropriate PROMs which provide a basis for further intervention planning.

Second, transplant professionals’ insights into correlations or determinants of psychosocial and behavioral or quality of life issues allows them to identify at-risk patients and to target modifiable determinants through targeted interventions. In this special issue, two studies describe barriers for medication adherence in heart transplant recipients. Denhaerynck et al. showed that primarily personal barriers, for example, sleepiness, being away from home and forgetfulness, were related to non-adherence to the immunosuppressive regimen. These findings were supported by the study of Marston et al., who showed that intentional non-adherence, i.e., recipients consciously deciding to reduce their dosing frequency or number of medications or discontinue treatment, is, alongside financial and accessibility barriers, mainly driven by personal barriers. Both studies show that multilevel determinants drive patient behavior pointing to the needs for multilevel interventions, i.e., not only targeting transplant recipients yet also healthcare provider, organization of care and healthcare system aspects.

Third, transplant professionals need to have knowledge about post-transplant outcomes that reflect how recipients feel and function, and their associated factors. Life participation, the ability to participate in meaningful activities of life, has found to be the most important outcome for kidney transplant recipients [1]. However, employment status, as part of life participation, has received limited attention. In a registry-based study, Mols et al. found that of the 40% of heart transplant recipients eligible for labor market participation, most (30%) were employed and 10% were unemployed. Unemployment was associated with multimorbidity and being socioeconomically disadvantaged. Although many transplant centers already address return to work as an important outcome post-transplant, the authors call for additional strategies to support workforce reintegration, particularly in those vulnerable groups.

Knowledge of patient-reported outcomes is also needed to support shared decision-making. Kidney transplantation, for example, is in general the treatment of choice for people with kidney failure as it offers better survival and quality of life compared with dialysis. The paper of De Boer et al. showed that this also applies to older (≥65 years) kidney transplant recipients as they perceived their physical and mental health-related quality of life as better when compared to older waitlisted kidney transplant candidates. This evidence can help older transplant candidates to make an informed decision whether to pursue a transplant or not.

Next to positive effects, potentially negative effects on outcomes should also be addressed. In their viewpoint article, Stylemans et al. describe the pros and cons of physical activity after transplantation. Although engaging in physical activity is beneficial for the health of transplant recipients, strenuous physical activity may come with potential adverse outcomes such as overuse injuries, increased risk of infections, and cardiovascular events. The authors state that the line between health benefits and potential harm of physical activity lies in the dosage administered, indicating that interventions to support recipients in living well posttransplant should always take the persons capabilities into consideration.

Lastly, it is important to evaluate if interventions or new models of care are effective in improving transplant outcomes, including those related to self-management and quality of life. The study of van Zanten et al. examined the effectiveness of a nurse-led, tailored intervention to promote self-management skills in solid organ transplant recipients. Although participants were positive about the program and reported added value, in terms of goal setting and providing tools to move forward after transplantation, the intervention was only effective for recipients with lower self-management skills at the start of the study. This indicates that a one-size fits all approach might not be effective and that it is important to identify transplant recipients who will benefit the most from an intervention based on certain characteristics, for example, health literacy, level of knowledge or skills, or demographic characteristics or economic status.

One specific aspect related to self-management is the monitoring of signs and symptoms by healthcare professionals during post-transplant follow-up care visits. Advancements in telemedicine and eHealth nowadays make it possible for transplant recipients to self-monitor their signs and symptoms in a reliable way at home. Hezer et al. studied the feasibility of implementing home-monitoring as standard care after kidney transplantation. The authors found that most kidney transplant recipients were open for home-monitoring, adhered to the protocol, were positive about the home-monitoring system and reported lower care needs due to home-monitoring. This study shows the potential of telemedicine and eHealth interventions in supporting self-management, especially in the light of the ever-growing transplant population. However, the effectiveness of home-monitoring still needs to be evaluated in a real-world setting with a focus on implementation in the clinical workflows.

Mielke et al. addressed another aspect of transplant care that is important when trying to achieve personalized care, a trustful relationship between transplant recipients and transplant professionals. Trust in the transplant team is gaining attraction as a relevant system level factor related to quality of care and as a determinant of health behavior. In their study, the authors found that heart transplant recipients who received care based on an integrated model of care, combined with longer consultation time and a chronic illness management approach, had greater trust in their transplant team, and showed better outcomes regarding dietary adherence. The authors conclude that trust and transplant care based on the principles of chronic illness management are key factors for reengineering transplant care aiming to optimize transplant outcomes.

In conclusion, based on the plea for person-centered transplant care made in this special issue by transplant recipients and transplant professionals, the time is ripe for reengineering transplant care. By providing care based on the needs and capabilities of transplant recipients, we can improve long-term outcomes after transplantation and enable transplant recipients to live well after transplantation. We hope the papers included in this special issue serve as a powerful source of inspiration for transplant programs across the globe.
